# Rapid Loss of Adiponectin-Stimulated Fatty Acid Oxidation in Skeletal Muscle of Rats Fed a High Fat Diet Is Not Due to Altered Muscle Redox State

**DOI:** 10.1371/journal.pone.0052193

**Published:** 2012-12-17

**Authors:** Ian R. W. Ritchie, David J. Dyck

**Affiliations:** Department of Human Health and Nutritional Sciences, University of Guelph, Guelph, Ontario, Canada; University of Texas Health Science Center at Houston, United States of America

## Abstract

A high fat (HF) diet rapidly impairs the ability of adiponectin (Ad) to stimulate fatty acid (FA) oxidation in oxidative soleus muscle, but the underlying mechanism remains elusive. Mere days of HF feeding also increase the muscle’s production and accumulation of reactive oxygen species (ROS) and shift cellular redox to a more oxidized state. It seems plausible that this shift towards a more oxidized state might act as negative feedback to suppress the ability of Ad to stimulate FA oxidation and generate more ROS. Therefore, we sought to determine whether i) a shift towards a more oxidized redox state (reduction in GSH/2GSSG) coincided with impaired Ad-stimulated palmitate oxidation in oxidative and glycolytic rodent muscle after 5 days of HF feeding (60% kCal), and ii) if supplementation with the antioxidant, N-acetylcysteine (NAC) could prevent the HF-diet induced impairment in Ad-response. Globular Ad (gAd) increased palmitate oxidation in isolated soleus and EDL muscles by 42% and 34%, respectively (p<0.05) but this was attenuated with HF feeding in both muscles. HF feeding decreased total GSH (−26%, p<0.05) and GSH/2GSSG (−49%, p<0.05) in soleus, but not EDL. Supplementation with NAC prevented the HF diet-induced reductions in GSH and GSH/2GSSG in soleus, but did not prevent the loss of Ad response in either muscle. Furthermore, direct incubations with H_2_O_2_ did not impair Ad-stimulated FA oxidation in either muscle. In conclusion, our data indicates that skeletal muscle Ad resistance is rapidly induced in both oxidative and glycolytic muscle, independently of altered cellular redox state.

## Introduction

Dysregulation of fatty acid (FA) metabolism and deposition of lipids represent one mechanism by which insulin response in skeletal muscle can be impaired [1], [2]. The adipose tissue-derived adipokine, adiponectin (Ad), reduces intramuscular lipid (IML) content and improves insulin sensitivity, in part through the stimulation of FA oxidation [3]. It is evident, however, that in insulin resistant conditions there is a resistance to Ad (i.e. a blunted stimulation of FA oxidation). Furthermore, this resistance can be induced in oxidative rodent muscle with the administration of a high saturated fat (HF) diet very rapidly i.e. within 3 days [4]. Given the dogma regarding Ad’s role as an insulin-sensitizing agent, we had hypothesized that the early induction of this resistance might be causative in the subsequent accumulation of muscle lipids and development of insulin resistance [4]. However, we have since observed that the restoration of muscle insulin response in HF fed rats occurs in the absence of any improvement in Ad response with interventions including training (under review) or fish oil supplementation [5]. These observations are not consistent with the hypothesis that early Ad resistance is an important contributor to insulin resistance in muscle.

Given how rapidly Ad resistance can be induced, it is conceivable that this phenomenon may actually be a protective mechanism, preventing excessive rates of FA entry into the mitochondrion. Adiponectin stimulates FA oxidation by increasing flux into the mitochondria secondary to stimulation of AMPK and a reduction in malonyl CoA production and its inhibition of CPTI. Interestingly, our findings [6], [7] and that of others [8] indicate that there is already a compensatory increase in muscle FA oxidation in rodents placed on a HF diet. Thus, it is conceivable that a resistance to Ad’s ability to further stimulate FA entry into mitochondria could serve to protect the mitochondria from excessive FAs and the generation of reactive oxygen species (ROS). To our knowledge, this possibility has not been considered.

Reactive oxygen species are a normal byproduct of energy metabolism and are an important regulatory sensor. The cellular redox environment is a reflection of the balance between ROS production and metabolism. The ratio of GSH (reduced glutathione) to GSSG (oxidized disulfide glutathione) is a reliable marker of cellular redox state [9] and is the most abundant redox couple in the cell. GSH serves as the cells primary redox buffer and also acts as dynamic regulator of redox sensitive enzymes [10], [11]. The function of numerous redox-sensitive proteins are altered by a shift in redox state, including stress kinases such as NFκB and Jun-kinase (JNK), as well as the insulin receptor substrate (IRS-1), leading to impaired insulin signaling [10], [11]. The activities of several protein tyrosine phosphatases are also highly dependent on the redox state of cysteine residues at their catalytic sites with obvious implications for multiple signal transduction pathways [12], [13].

The GSH/2GSSG ratio and total GSH content are decreased in muscle from obese individuals (i.e. shift towards more oxidized state), as well as in rodent muscle following a 6-week HF diet [14]. Intriguingly, Anderson et al. also reported that a 5-day HF diet in humans can reduce muscle total GSH and shift the redox to a more oxidized state [14]. This time frame is remarkably similar to that in which Ad’s ability to stimulate FA oxidation is impaired by a HF diet in rodents. We therefore speculated that a rapid change in cellular redox might be responsible for the HF-diet induced development of Ad resistance in rat muscle.

The purpose of this study was to determine i) if a brief period (i.e. 5 days) of HF feeding could result in a more oxidized redox state (oxidative stress) in rat skeletal muscle that coincided with the rapid loss of Ad’s ability to stimulate FA oxidation; ii) if the HF-diet induced Ad resistance could be prevented through supplementation with the general anti-oxidant N-acetylcysteine (NAC); and iii) to examine and compare these responses in muscles of different oxidative capacity (oxidative (soleus) and glycolytic (extensor digitorum longus, EDL)).

## Methods

### Ethics Statement

All procedures were carried out in accordance with the recommendations of the Canadian Council of Animal Care, and were approved by the Animal Care Committee at the University of Guelph (Animal Utilization Protocol 11R017). All surgeries were performed under sodium pentobarbital anesthesia and all efforts made to prevent discomfort and suffering.

### Animals and Diets

Female Sprague Dawley rats (60 g; Charles River, St Constant, QC) were housed in groups on a 12-hour reverse-dark cycle and fed standard rodent chow. Animals were given 48 hours to acclimate to their environment before being assigned to their respective experimental groups. Animals were randomly assigned to one of two diets. Half of the animals maintained their *ad libitum* chow diet (CON; Harlan Teklad, Madison, WI) while the other half were give *ad libitum* access to a diet high in saturated fat (HF; 60% total kCal; Research diets, New Brunswick, NJ) with the purpose of inducing muscle Ad resistance. Within each dietary group, half of the animals were given the antioxidant, N-acetylcysteine (NAC; 10 mM in drinking water), for a total of 4 experimental groups (CON, CON+NAC, HF, HF+NAC; n = 12 per group). Dietary/experimental treatments lasted for 5 days at which time animals were sacrificed following an 8 to 12-hour overnight fast.

### Tissue Sampling

Following an overnight fast, animals were anaesthetized with an intraperitoneal injection of sodium pentobarbital (6 mg/100 g body wt). Soleus (oxidative) and EDL (glycolytic) muscles from one hind limb were stripped longitudinally and excised, yielding two intact muscle strips which were used to assess basal and Ad-stimulated palmitate oxidation (soleus strips, 16.4±0.4 mg; EDL strips, 20.6±0.4 mg). Whole soleus and EDL muscles were removed from the second leg and flash frozen in liquid nitrogen for the assessment of total and oxidized glutathione content. Red and white gastrocnemius muscles were sampled and flash frozen for the assessment of protein carbonylation as a marker of oxidative damage. In a separate set of experiments, soleus and EDL muscle strips from chow-fed animals were incubated in medium containing 0, 0.5 or 2 mM H_2_O_2_ to determine whether there was a direct effect of altered muscle redox state on Ad’s ability to stimulate FA oxidation.

### Basal and Ad-stimulated Palmitate Oxidation

Pre-warmed (30°C), pre-gassed (95%O_2_/5%CO_2_) Medium 199, containing 4% BSA and 0.5 mM palmitate was used as a base for all buffers. Immediately following isolation, soleus and EDL muscle strips were placed in pre incubation buffer for 30 min. This buffer consisted of the base buffer only. Following pre-incubation, muscle strips were carefully transferred to vials containing incubation buffer for 60 min. The incubation buffer consisted of the base buffer with the addition of 0.5 uCi/mL [1-^14 ^C] palmitate (GE Healthcare; Baie d’Urfe, Que). In the Ad-stimulated conditions, gAd (murine recombinant, E. coli source; Peprotech, Dollard des Ormeaux, QC) was added for a final concentration of 2.5 ug/mL, which is the concentration previously used to elicit a max response of FA oxidation [15].

Following incubation, the muscle strips were removed and blotted, trimmed of their tendons and weighed. They were then placed into 5 mL of ice-cold 2∶1 chloroform: methanol in a 13 mL centrifuge tube. One mL of incubation buffer from each vial was immediately transferred to a sealed 50 mL Erlenmeyer flask and acidified with 1 mL of 1 M sulfuric acid. Liberated ^14^CO_2_ was captured in 250 uL of 1 M benzethonium hydroxide suspended in a 500 uL tube within the flask. This tube was then counted using a liquid scintillation counter.

Muscle samples were homogenized using a Brinkman Polytron at 25 000 rpm, 2 mL of Milli Q water was added to separate aqueous and lipid soluble phases, and samples were placed on a rocker for 10 minutes. Following the extraction, 0.5 mL of the aqueous phase was sampled in duplicate and counted using a liquid-scintillation counter to measure ^14^C trapped in citric acid cycle intermediates (isotopic fixation). Palmitate oxidation was interpreted as the sum of that from the benzathonium hydroxide and the aqueous extract.

In a separate set of experiments, basal and Ad-stimulated FA oxidation was measured in soleus and EDL muscle strips following incubation in medium containing 0, 0.5 or 2 mM H_2_O_2_ for 2 hours to determine whether there was a direct effect of altered muscle redox state on Ad response. In pilot experiments, we determined that the acute exposure to 0.5 mM H_2_O_2_ for 2 hours increased the content of oxidized glutathione and decreased the ratio of total to oxidized glutathione ratio by about 50%, which is similar to that achieved by 5 days of HF feeding. Palmitate oxidation was assessed immediately following the two hour incubation.

### Glutathione and Protein Carbonyls

Total and oxidized glutathione content were assessed in soleus and EDL muscles removed from fasted animals to determine the effects of diet and antioxidant supplementation. Samples were powdered under liquid nitrogen and split into two tubes containing 5% metaphosphoric acid. To assess oxidized GSH, methyl-2-vinylpyridinium triflate was added to rapidly remove reduced GSH. Following a 5 min incubation, samples were centrifuged at 1 000×*g* for 10 min and the supernatant was removed and analyzed using a commercially available kit (Cat. No. 21040; Oxis International, Beverly Hills, CA). Concentrations were expressed per gram of extract protein as determined by BCA assay. In a separate set of incubations, we analyzed total and oxidized glutathione content in soleus and EDL muscle strips after a 30 min incubation in base buffer to ensure that dietary affects on muscle redox state were still present (n = 5 per group).

Protein carbonyls were measured in other representative oxidative and glycolytic muscle (red and white gastrocnemius), due to the fact that the smaller soleus and EDL muscles were completely utilized for the assessment of palmitate oxidation and glutathione content. Briefly, muscle was chipped under liquid nitrogen and homogenized in 200 uL of 0.1 M PBS containing a protease inhibitor cocktail (Cat. No. P8340; Sigma Aldrich). Samples were centrifuged at 1500×*g* and the supernatant assayed for total protein and diluted in PBS to a final protein concentration of 10 ug/mL. Protein carbonyls were then measured using a commercially available ELISA kit (Cat. No. STA-310; Cell Biolabs, San Diego, CA).

### Statistical Analysis

All data are reported as mean ± the standard error (SE). Ad-stimulated FA oxidation, glutathione and protein carbonyl data following HF feeding and/or NAC treatment, was analyzed using a randomized block-design two-way (diet, antioxidant) ANOVA. Ad-stimulated FA oxidation data following H_2_O_2_ treatment was analyzed using a paired t-test. Statistical significance was accepted at p≤0.05.

## Results

### Body Weight and Fasting Blood Glucose

There were no significant differences in terminal body weight or fasting blood glucose among the experimental groups (Table 1).

**Table 1:** Terminal body weights and fasting blood glucose.

	CON	CON+NAC	HF	HF+NAC
**Terminal** **body wt,** **grams**	107±1	104±2	110±5	114±2
**Fasting blood** **glucose mM**	7.7±0.3	8.1±0.4	9.0±0.3	9.4±0.4

Data are expressed as means +/− SE, n = 8–12. CON, control diet; CON+NAC, control diet+N-acetylcysteine (antioxidant); HF, high fat diet; HF+NAC, high fat diet+N-acetylcysteine (antioxidant).

### FA Oxidation

**Figure 1:**
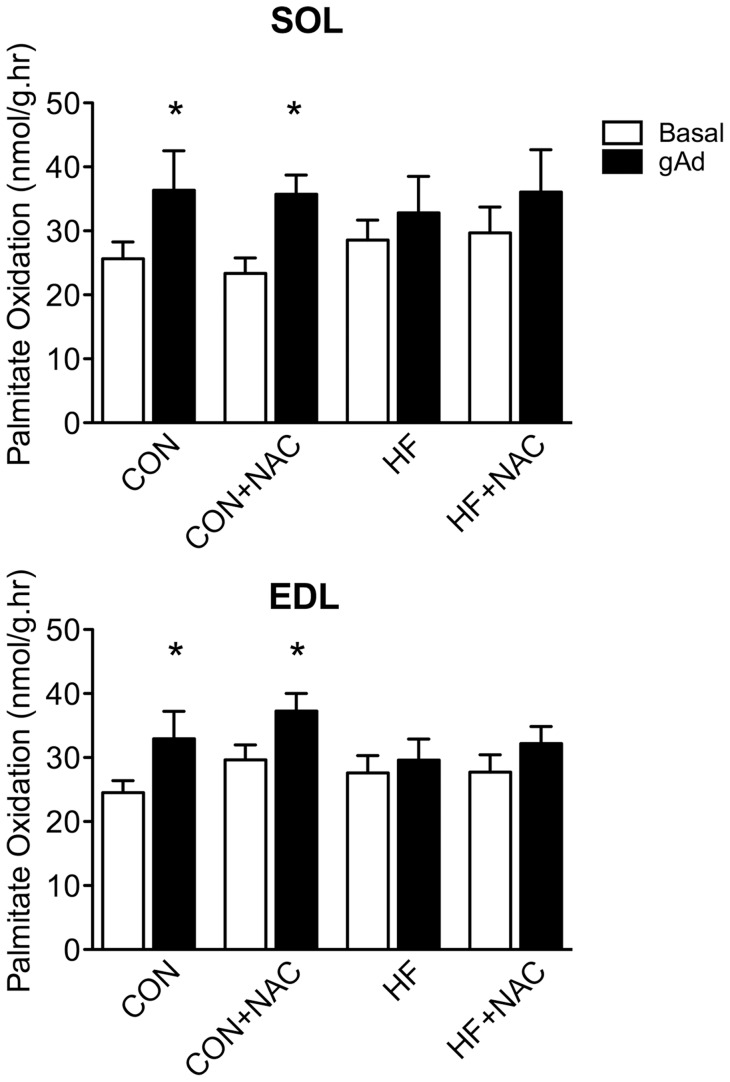
Palmitate oxidation in isolated soleus (A) and extensor digitorum longus (B) muscle strips in the presence and absence of adiponectin. Basal, empty bars; gAd-stimulated, filled bars. Data are expressed as means+SE, n = 8–12. * denotes significant difference from respective basal condition, P≤0.05. Muscles were isolated from animals in the 4, 5-day dietary interventions (CON, control diet; CON+NAC, control diet+N-acetylcysteine (antioxidant); HF, high fat diet; HF+NAC, high fat diet+N-acetylcysteine (antioxidant)) for 60 minutes.

**Figure 2:**
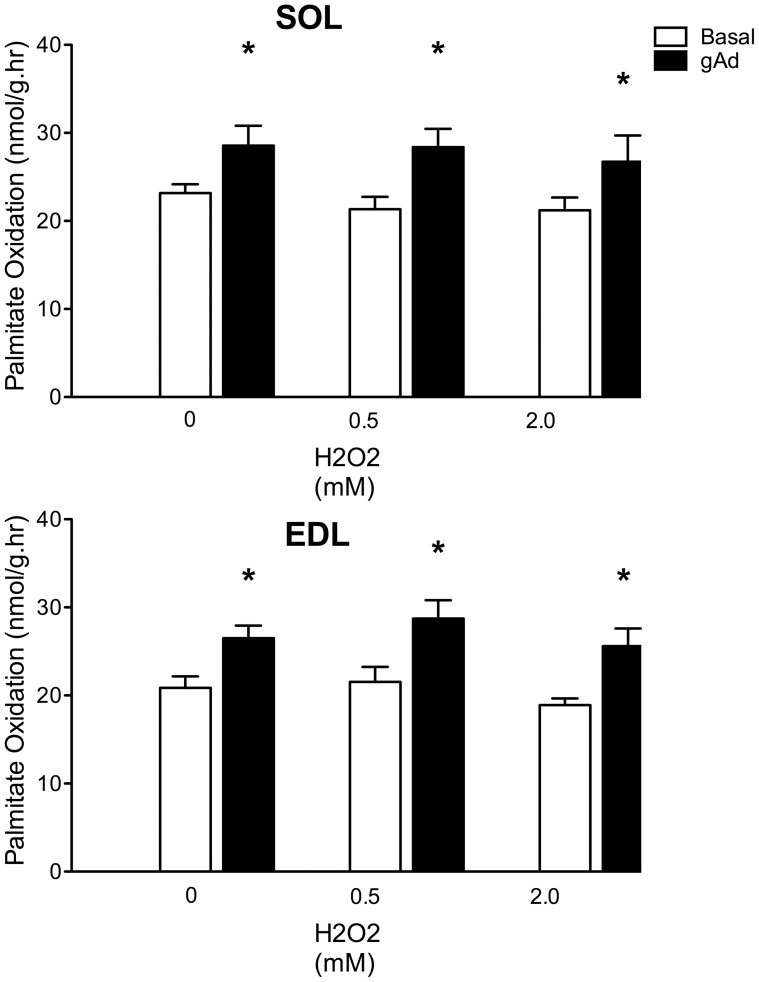
Ability of adiponectin to stimulate palmitate oxidation after incubation with varying concentrations of hydrogen peroxide. A) Soleus muscle strips; B) extensor digitorum longus strips. Basal (empty bars); gAd-stimulated (filled bars). Data are expressed as means+SE, n = 8–12. * denotes significant difference from respective basal condition, P≤0.05. Muscles were isolated from control (chow-fed) animals and incubated for 120 minutes at 0, 0.5 or 2.0 mM H_2_O_2_. Palmitate oxidation was then assessed.

Incubation with Ad increased palmitate oxidation in soleus and EDL by 42 and 34% respectively (p<0.05) in control rats (Figure 1). There was no significant increase in palmitate oxidation in either muscle in response to Ad following 5 days of HF feeding. Treatment with NAC alone had no effect on Ad-stimulated FA oxidation. The concurrent administration of NAC with a HF diet failed to improve or restore the ability of Ad to stimulate palmitate oxidation. Acute incubations with 0.5 and 2.0 mM H_2_O_2_ did not impair the ability of Ad to stimulate FA oxidation in either muscle (Figure 2).

### Glutathione Content

**Figure 3:**
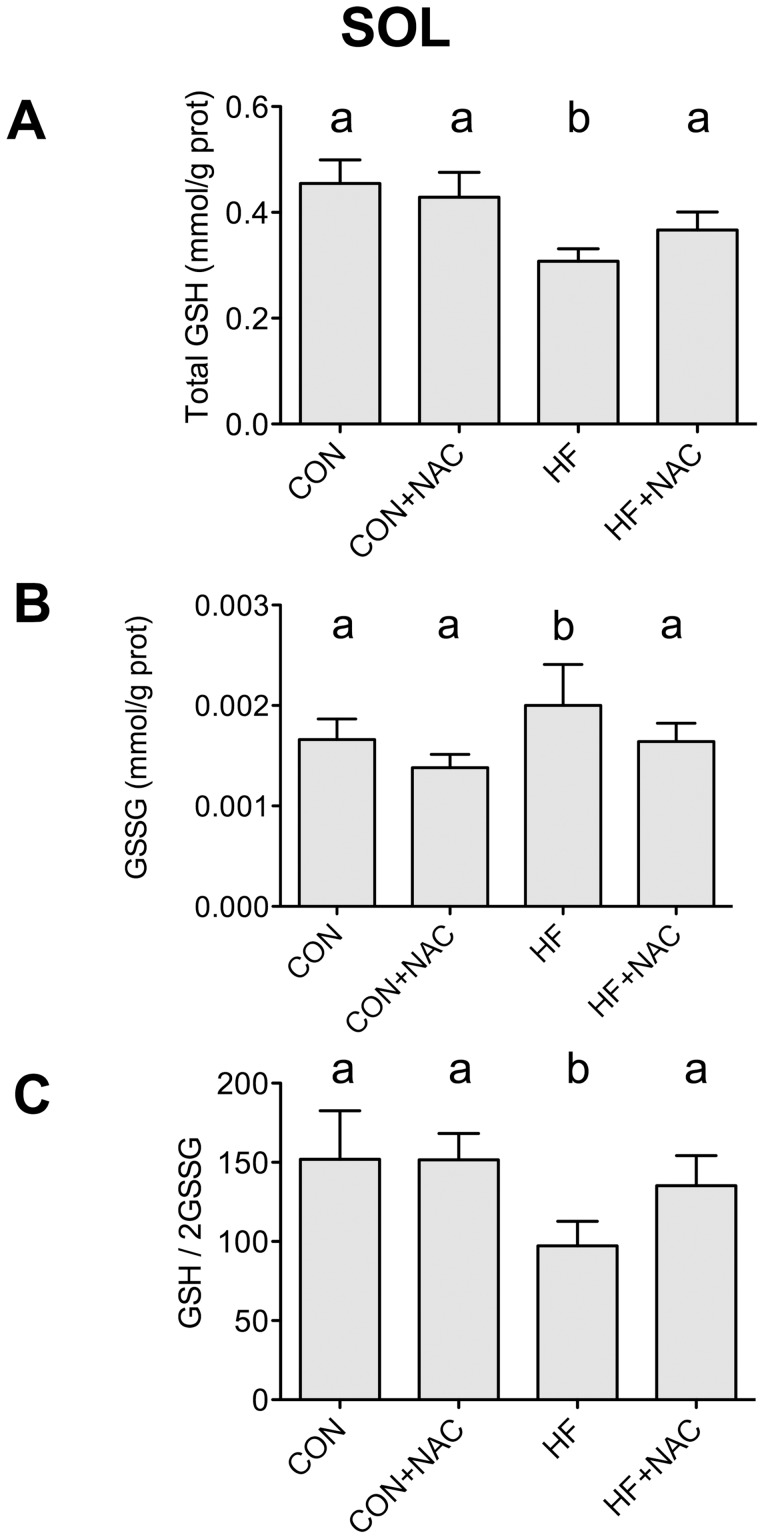
Content of total glutathione (A), oxidized glutathione (B), and the ratio of oxidized to reduced glutathione (C) from soleus muscle. Data are expressed as means+SE, n = 8–12. Bars not sharing a letter are significantly different, P≤0.05. Muscles were removed from animals on the 4 dietary interventions (CON, control diet; CON+NAC, control diet+N-acetylcysteine (antioxidant); HF, high fat diet; HF+NAC, high fat diet+N-acetylcysteine (antioxidant)).

The content of total glutathione (GSH) in soleus muscle from animals fed a HF diet was reduced by 26% compared to that of control-fed animals (p<0.05; Figure 3A). High fat feeding increased oxidized glutathione (GSSG) content in soleus muscle 46% (p<0.05; Figure 3B) and significantly reduced the GSH/2GSSG ratio (−49%, p<0.05; Figure 3C). Each of these HF diet-induced effects on total and oxidized glutathione was prevented by concurrent supplementation of the HF diet with NAC. Supplementation with NAC alone had no effect on soleus total and oxidized glutathione content, or the ratio between the two. Following a 30 min incubation, the ratio of reduced to oxidized glutathione (GSH/2GSSG) in isolated soleus muscle was 48% lower (p<0.05) in the HF group relative to CON; the ratios in incubated solei from NAC and HF+NAC conditions remained similar to CON (data not shown). Thus, diet-induced differences were preserved.

**Figure 4:**
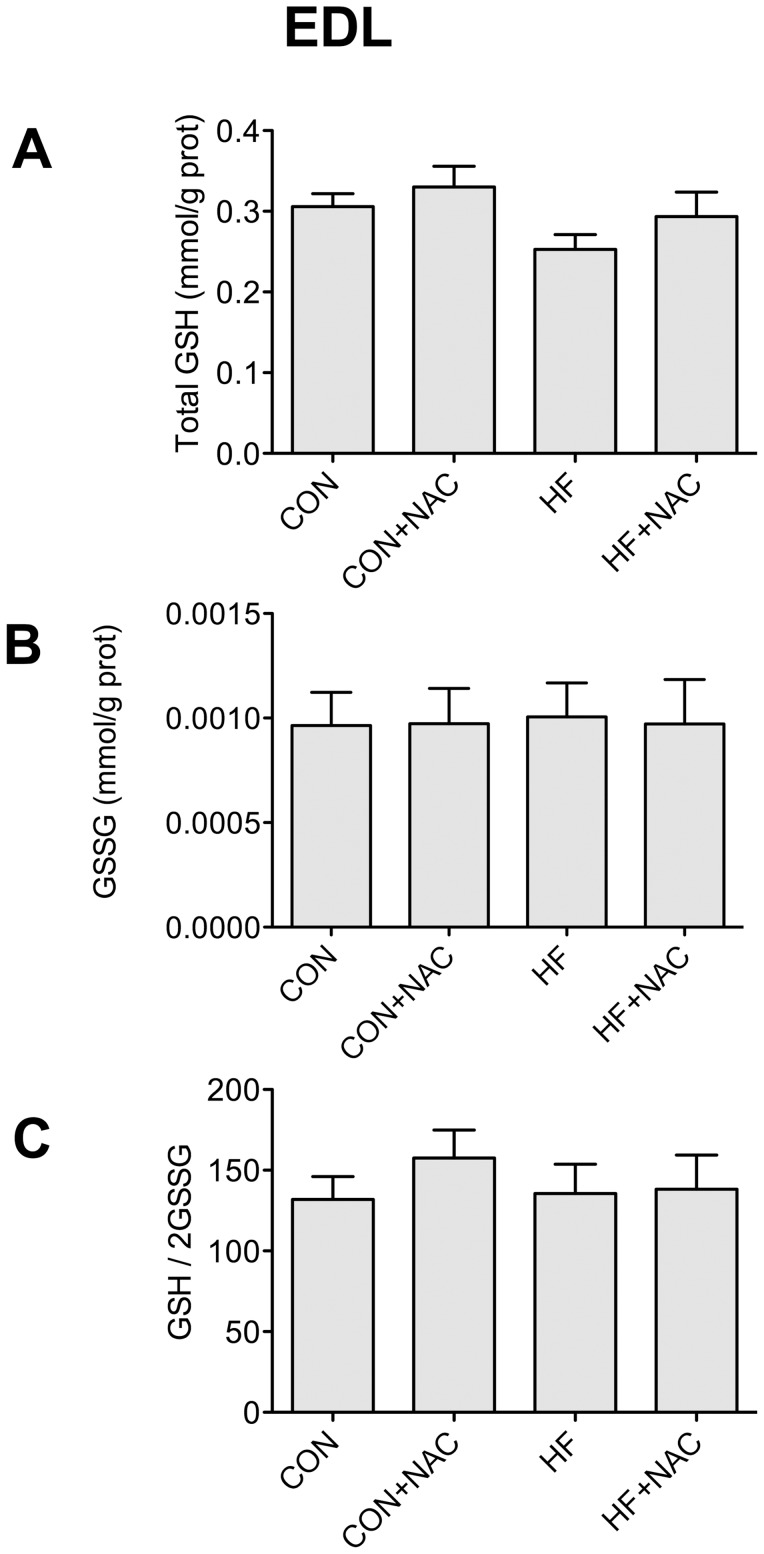
Content of total glutathione (A), oxidized glutathione (B), and the ratio of oxidized to reduced glutathione (C) from extensor digitorum longus muscle. Data are expressed as means+SE, n = 8–12. Bars not sharing a letter are significantly different, P≤0.05. Muscles were removed from animals on the 4 dietary interventions (CON, control diet; CON+NAC, control diet+N-acetylcysteine (antioxidant); HF, high fat diet; HF+NAC, high fat diet+N-acetylcysteine (antioxidant)).

Glutathione content (total, oxidized, ratio) was not affected in EDL with either HF feeding or NAC supplementation (Figure 4).

### Protein Carbonylation

**Figure 5:**
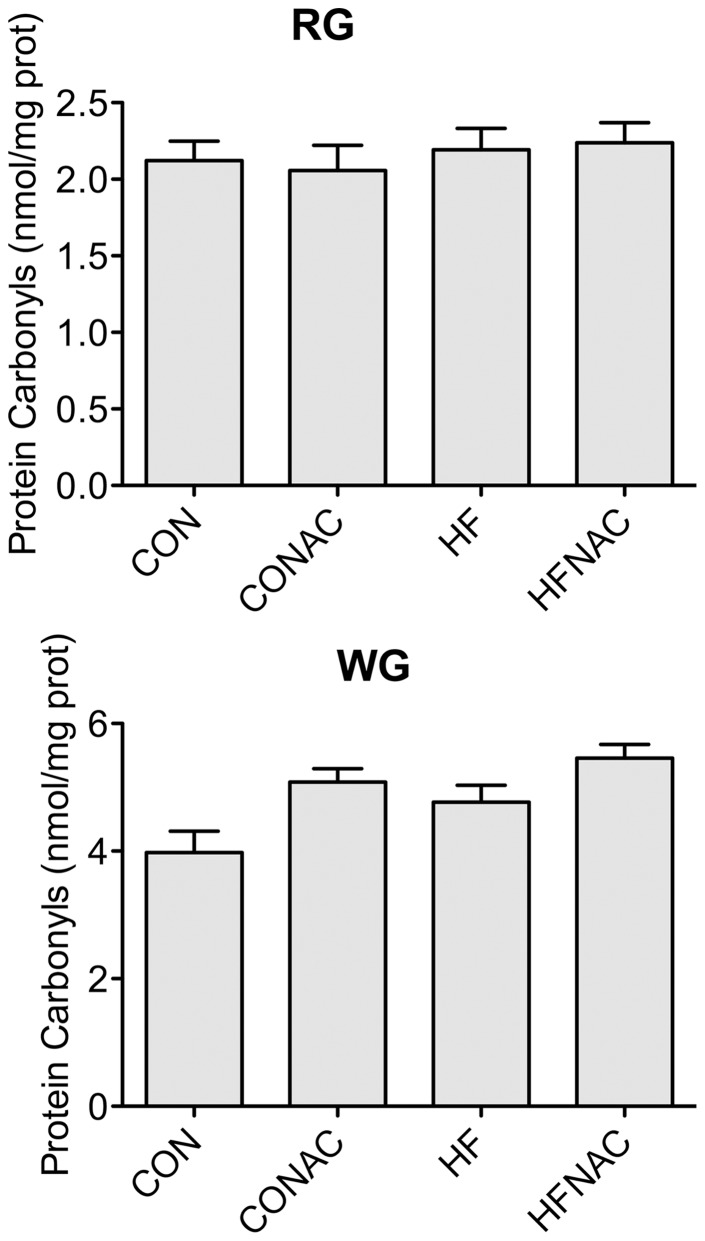
Content of protein carbonyls from red (A) and white (B) gastrocnemius. Data are expressed as means+SE, n = 8–12. *denotes significant difference, P≤0.05. Muscles were removed from animals on the 4 dietary interventions (CON, control diet; CON+NAC, control diet+N-acetylcysteine (antioxidant); HF, high fat diet; HF+NAC, high fat diet+N-acetylcysteine (antioxidant)).

High fat feeding had no significant effect on protein carbonylation in red and white gastrocnemius muscle. Supplementation with NAC also had no significant effect on protein carbonylation in either muscle type (Figure 5).

## Discussion

The ability of Ad to stimulate FA oxidation is blunted in muscle from obese humans and in HF-fed rodents [4], [16]. Surprisingly, very little is known regarding the underlying mechanism by which Ad response is impaired. Intriguingly, changes in the cellular redox environment have been found to occur following mere days of HF feeding [14], similar to the time frame in which a HF diet can induce Ad resistance in rodents [4]. Alterations in the redox state have significant effects on cellular signaling [10], [11], [12], [13] and could potentially be causative in the disruption of Ad signaling. In the current study, we demonstrate that 5 days of HF feeding impairs Ad-stimulated palmitate oxidation in both oxidative and glycolytic skeletal muscle. This loss of Ad response was accompanied by a decrease in the GSH/2GSSG ratio and total GSH content in oxidative, but not in glycolytic muscle. Supplementation with the general antioxidant NAC prevented the decrease in GSH/2GSSG ratio and GSH depletion in soleus, but did not protect against the loss of Ad response in either fiber type. Direct incubation with H_2_O_2_ altered redox state in both muscles, but did not impair the ability of Ad to stimulate FA oxidation. Neither HF feeding nor NAC supplementation had a discernable effect on protein carbonylation in either fiber type. Collectively, our data indicates that the rapid onset of Ad resistance occurs independently of changes in the redox state.

### HF Feeding Impairs Ad Response in Both Fiber Types, but only Alters Redox Status in Soleus Muscle

The insulin sensitizing effects of Ad are believed to be due in part to the stimulation of FA oxidation [3]. This response is impaired in obesity and by as little as 3 days of HF feeding in rodents [4], [6]. Although it is known that Ad can stimulate FA oxidation in both muscle fiber types [17], it was not known prior to the current study that the rapid of loss of Ad-stimulated FA oxidation extends to both fiber types. It has previously been our belief that the rapid onset of Ad resistance may contribute to subsequent development of insulin resistance in muscle. While this is indeed a possibility, our more recent findings that the rapid reversal of diet-induced insulin resistance does not require the restoration of response to either leptin [18] or adiponectin [5] challenges this belief. Given that the rapid development of Ad resistance with a HF diet appears to coincide with increased ROS production, led to our current view that Ad resistance may initially be a protective mechanism to prevent excessive FA entry into the mitochondria. However, any such potential benefit to Ad resistance does not imply that this is desirable in the long term, as previous studies have clearly demonstrated that disruption of AdipoR1 can lead to inflammation and oxidative stress [19], [20].

GSH scavenges oxidants by oxidation of the central cysteine moiety at which point it can form glutathione disulfide (GSSG) or mixed disulfides with other cysteine containing proteins. In this way GSH can also act as a substrate in the regulation of redox sensitive proteins [10], [11]. In either case, oxidized glutathione is reduced and GSH can be regenerated by glutathione reductase [21]. The ratio of GSH/2GSSG is a reflection of the balance between oxidant production and enzymatic reduction and defines the redox environment in the cytosol [9]. In this study, we report that 5 days of a HF diet is sufficient to decrease the ratio of reduced to oxidized GSH (GSH/2GSSG) in oxidative soleus muscle indicating a shift to a more oxidized state. There was also a significant decrease in the total GSH content of soleus, indicating a decreased capacity to buffer oxidants. This is consistent with previous findings in human muscle after 5 days of HF feeding [14] or with rodents after 6 weeks of HF feeding [22]. As far as we are aware, this is the first study to report a change in redox state and buffering capacity in rodent muscle within a time frame as short as 5 days.

In the current study, we did not observe a diet effect on the redox state in glycolytic skeletal muscle (EDL). Recently, ROS generation in isolated mitochondria has been shown to be elevated when respiration is supported predominantly by long chain FA [23]. In addition, lipid infusions have been found to acutely increase mitochondrial ROS production and reduce total GSH and GSH/2GSSG in gastrocnemius muscle, which contains a large portion of oxidative fibers [24]. These effects can be prevented with the coinfusion of NAC [24]. However, glycolytic muscle has a reduced expression of FA transporters and rates of FA transport across both the sarcolemmal and mitochondrial membranes [25], [26]. Therefore it is possible that glycolytic muscle is inherently less susceptible to a HF diet, precluding our ability to significantly decrease the GSH/2GSSG ratio and total GSH content in this fiber type.

Prolonged changes in redox state and depressions in oxidant buffering capacity increase the likelihood for oxidative protein damage [27]. We found no changes in protein carbonylation following HF feeding with or without NAC supplementation, unlike others who have reported diet-induced increases in protein carbonylation following a 16-week high fat, high sucrose diet [22]. However, comparison is difficult due to discrepancies in both dietary composition and study duration. It is possible that a longer protocol would have yielded a change in protein carbonylation in the present study.

### Antioxidant Supplementation does not Protect Ad Response

N-acetylcysteine is an acetylated derivative of the amino acid cysteine, the rate limiting substrate in GSH production [28]. NAC is also readily oxidized, thereby acting as an antioxidant in much the same way as GSH. NAC has been shown to prevent oxidative stress in a number of different experimental models including muscle cell incubations with palmitate or H_2_O_2_, muscle from streptozotocin-treated mice [22], and exercise in both humans and rats [29], [30]. Here, we report that NAC supplementation to HF fed rodents prevented the reductions in total GSH and the GSH/2GSSH ratio in soleus. Importantly, however, this maintenance of redox state and antioxidant capacity was not accompanied by the preservation of Ad-stimulated palmitate oxidation. Therefore, in this study, we have divorced HF diet-induced shifts in cellular redox state and Ad resistance in two ways. First, we show an impaired Ad response in EDL following 5 days of HF feeding without any significant effects on total GSH or redox state. Second, we demonstrate that maintaining cellular redox state by supplementation with NAC does not prevent the impairment in Ad response in soleus muscle. Finally, directly altering redox state in both soleus and EDL muscles, via incubation with H_2_O_2_, did not impair Ad-stimulated FA oxidation.

It should be recognized that while NAC is effective at preventing oxidative stress in several experimental models [22], [24], [30], the subcellular source of ROS generation can vary and cellular antioxidants are also regionalized [31], [32]. These specific regional contributions would not be accurately represented in a whole muscle homogenate as assessed in the current study. Furthermore, redox sensitive proteins have different redox potentials and therefore different degrees of susceptibility to this type of regulation. Therefore, it is entirely possible that these regulatory events are occurring at a level below what is realistically measurable by assessing GSH and general protein carbonylation in whole muscle extracts.

### Perspectives and Conclusions

We have previously reported that the ability of Ad to stimulate FA oxidation in soleus is impaired within 3 days of HF feeding [4]. Interestingly, plasma adiponectin is also decreased within this 3 day period [4]. However, the cause of rapidly induced Ad resistance has not yet been elucidated. We have previously shown in soleus that there is no change in the protein content of AdipoR1 [4], APPL1 [5] and APPL2 (Gulli et al, in press Am J Physiol Reg) in response to HF diets lasting at least 4 weeks. Muscle lipids have been shown to increase by 2 weeks, but not after 3 days of HF feeding [4]; therefore, we cannot rule out the possibility that they were elevated by 5 days in the present study. However, even if this was the case, baseline rates of FA oxidation were unaffected after 5 days of HF feeding.

The results from our current study further our previous work by demonstrating that Ad resistance develops rapidly in both oxidative and glycolytic muscle fiber types. The redox state of the oxidative soleus muscle was altered within the same time frame as the development of Ad resistance; however, the redox state was unaltered in glycolytic muscle. Furthermore, while treatment with the antioxidant NAC prevents HF diet-induced alterations in redox state in the soleus, this did not result in the protection of Ad response. From these findings we must conclude that shifts in whole muscle redox state are not responsible for the rapid impairment in Ad response.
